# Alzheimer's Disease‐Related CSF Biomarker Patterns and Clinical Characteristics in PSP and CBS

**DOI:** 10.1002/alz70856_104671

**Published:** 2026-01-07

**Authors:** Takanobu Ishiguro, Kensaku Kasuga, Tamao Tsukie, Osamu Onodera, Takeshi Ikeuchi

**Affiliations:** ^1^ Brain Research Institute, Niigata University, Niigata, Japan

## Abstract

**Background:**

To investigate the impact of cerebrospinal fluid (CSF) biomarker profiles, specifically amyloid‐positive (A+) and Alzheimer's disease (AD) pattern (A+T+), on the clinical characteristics of progressive supranuclear palsy (PSP) and corticobasal syndrome (CBS).

**Method:**

We studied patients diagnosed at our institution with PSP based on the Movement Disorders Society criteria (2017) or CBS based on Armstrong's criteria for corticobasal degeneration (2013), meeting either possible or probable levels of diagnostic certainty. CSF biomarkers were measured for AD‐related markers, including the Aβ42/Aβ40 ratio (A marker), phosphorylated tau (*p*‐tau181, T marker), and neurofilament light chain (*N* marker). Positivity for each biomarker was determined using our institutional cutoff values. Patients were classified as A+/A− or AD pattern (A+T+) versus non‐AD pattern, and their clinical characteristics were compared.

**Result:**

We analyzed 20 PSP cases (median age at onset: 69.5 years, 8 females) and 22 CBS cases (median age at onset: 65.5 years, 14 females). A+ biomarker profiles were observed in 12 PSP cases (60%) and 7 CBS cases (32%). AD pattern PSP was identified in 4 cases (20%), whereas AD pattern CBS was observed in 5 cases (23%). PSP patients with A+ biomarker profiles were significantly older at both disease onset and at the time of evaluation (*p* = 0.0013, *p* = 0.0022). Both PSP‐AD and CBS‐AD cases exhibited shorter disease durations from onset to evaluation (*p* = 0.032, *p* = 0.039). CBS‐AD cases demonstrated lower MMSE scores (*p* = 0.0368) and presented with right upper/lower limb symptoms (*p* = 0.0065). Additionally, they exhibited Gerstmann syndrome combined with naming difficulties, characteristic of angular gyrus syndrome (*p* = 0.007).

**Conclusion:**

In PSP, coexisting amyloid pathology appears to be age‐related. AD pattern CBS was characterized by apraxia in the right upper/lower limbs due to left hemispheric dysfunction, as well as angular gyrus syndrome, including Gerstmann syndrome and naming difficulties.

